# A 105 kb interstitial insertion in the Xq27.1 palindrome from pseudoautosomal region PAR1 causes a novel X-linked recessive compound phenotype

**DOI:** 10.1186/s12967-019-1887-2

**Published:** 2019-04-29

**Authors:** Nuo Si, Xiaolu Meng, Zhen Zhao, Weibo Xia, Xue Zhang

**Affiliations:** 1 McKusick-Zhang Center for Genetic Medicine, State Key Laboratory of Medical Molecular Biology, Institute of Basic Medical Sciences Chinese Academy of Medical Sciences, School of Basic Medicine Peking Union Medical College, Beijing, 100005 China; 2 Department of Endocrinology, Key Laboratory of Endocrinology, Ministry of Health, Peking Union Medical College Hospital, Chinese Academy of Medical Sciences, Beijing, 100730 China

**Keywords:** Interstitial insertion, Pseudoautosomal region 1, Xq27.1 palindrome, X-linked recessive, Genu varum

## Abstract

**Background:**

Genomic disorders present a wide spectrum of unrelated clinical entities that result from genomic rearrangements. Interstitial insertions requiring three points of breakage are rare genomic rearrangement events. The pseudoautosomal region PAR1, homologous between the Xp22 and Yp11 loci, has a high crossover and recombination rate. A 180 bp human-specific palindrome at Xq27.1 appears to be a hotspot for genomic rearrangement, and several genetic diseases/phenotypes associated with Xq27.1 palindrome-driven genomic rearrangement have been reported. Here we investigate a Chinese family with an extremely rare X-linked compound phenotype that remains undiagnosed. We attempt to identify underlying genetic causes by an integrated genome analysis.

**Methods:**

A five-generation Chinese family with a distinct X-linked compound phenotype was recruited. Peripheral blood samples were collected and genomic DNA was extracted. Systemic physical and lab examinations were performed to evaluate the phenotype. An integrated genomic analysis was performed. Genotyping and linkage analysis were conducted to map the disease locus. Whole exome sequencing was performed to detect mutations in coding region. Whole genome sequencing was used to detect single nucleotide variations, small insertions, small deletions, or large structural variations. Copy number variation scanning was also performed on the genome scale. Interstitial insertion was confirmed by gap-PCR and quantitative-PCR, and breakpoint junctions were identified by genome walking and direct sequencing. Expression of products of genes nearby to the Xq27.1 palindrome was measured in peripheral blood from patients and unrelated controls via quantitative-PCR.

**Results:**

The identified compound phenotype of genu varum, cubitus valgus, and everted lipsdoes not match any reported clinical entities. Fine mapping and linkage analysis identified a candidate interval of 4 Mb on the X chromosome. No potential coding region mutations were detected. A 105 kb genomic fragment of PAR1 containing no coding genes was duplicated and inserted into the center of a human-specific palindrome at Xq27.1. The interstitial insertion fully cosegregated with the family phenotype. No expression of *FGF13* or *SOX3* was detected in peripheral blood from the proband or unrelated controls.

**Conclusion:**

We report an extremely rare phenotype associated with an infrequently-seen genomic rearrangement. The novel compound phenotype is X-linked and characterized by genu varum, cubitus valgus, and everted lips. A 105 kb interstitial insertion of a PAR1 fragment into the Xq27.1 palindrome is associated with the phenotype in the family. The present study identified the underlying genetic cause of the phenotype, expanding the spectrum of known human-specific Xq27.1 palindrome insertion events and associated phenotypes.

**Electronic supplementary material:**

The online version of this article (10.1186/s12967-019-1887-2) contains supplementary material, which is available to authorized users.

## Background

With the efforts of the medical and science communities and the revolutionary progress made in the field of DNA analysis, more than 5000 mendelian disorders are now well recognized both by phenotype and molecular basis [1]. However, thousands of rare genetic diseases remain undiagnosed [1]. Some have described phenotypes with unknown molecular basis, while others are only known as suspected mendelian basis. For these conditions, no diagnoses are available, no biological causes are understood, and progression or whether a therapy can be found is unknown. We collected a Chinese family with extremely rare phenotypes transmitted in an X-linked recessive manner. Ten patients from the family were all male and present with distinctive compound phenotypes including genu varum, cubitus valgus, and everted lips. The compound phenotypes have profound adverse effects on the patients’ daily lives. No diagnosis was available, and no treatment was effective even with surgical intervention.

Genomic disorders account for a wide spectrum of unrelated clinical entities resulting from genomic rearrangements [2]. A growing number of genomic disorders have been recognized due to detection of complex rearrangements at high resolution with advanced genome technologies. Genomic rearrangements involve gross alterations of chromosomes or large genomic segments from a few kilobases (kb) to several megabases (Mb) in length. They may take the form of deletions, duplications, insertions, inversions, translocations, or complex rearrangements combining different forms. Interstitial insertions require three breaks in the genome and are estimated to be at least tenfold less frequent than two-break rearrangements such as duplications and deletions [3].

Genomic rearrangements are associated with specific genomic architectural features such as low copy repeats (LCRs) and palindromes. A unique 180 bp palindrome exists at Xq27.1 flanked by long interspersed elements-1 (LINE1) and long terminal repeat (LTR) sequences [4]. This distinct sequence feature makes this genomic region highly unstable and a hotspot for genomic rearrangement. Several interstitial insertions mediated by the Xq27.1 palindrome and their associated human diseases/phenotypes have been reported [4–9].

Pseudoautosomal regions (PARs) are short regions of homology between the mammalian X and Y chromosomes [10]. PAR1 and PAR2 reside at either end of the X and Y chromosomes, respectively. PAR1 is at the tips of the short ‘p’ arms (Xp22 and Yp11) spanning 2.6 Mb, while PAR2 is at the tips of the long “q” arms (Xq28 and Yq12) with a smaller size of 320 kb. PARs are required for pairing of the X and Y chromosomes and behave like autosomes during male meiosis. Because of its larger size, PAR1 supports obligate crossovers and has a crossover rate 17-fold greater than the genome-wide average [11]. However, PAR1-linked copy number variations (CNVs) are rarely reported and remain poorly investigated [12–14].

Here we report an extremely rare X-linked recessive phenotype and performed anintegrated genomic analysis in the family. An rare 105 kb interstitial insertion from PAR1 into the Xq27.1 human-specific palindromic sequence was identified and fully-cosegregated in the family. The results provide an etiological diagnosis for the family and suggest that the rare X-linked phenotype results from a genomic disorder.

## Materials and methods

### Subjects

**Fig. 1:**
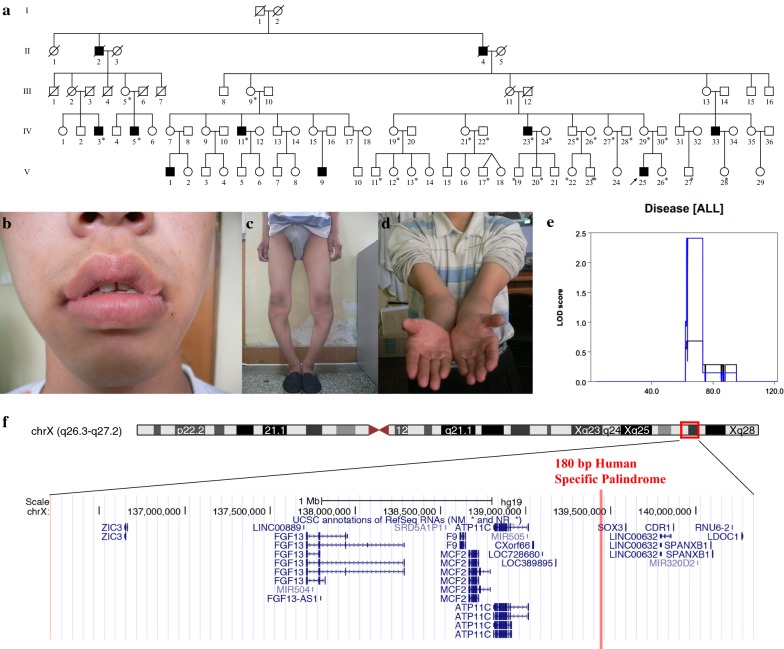
Phenotypes and genetic locus of a Chinese family with rare X-linked compound phenotypes. **a** Pedigree of the family with the X-linked recessive phenotype. Individuals with peripheral blood samples available are indicated by “*”, arrow indicates the proband. **b**–**d** Compound phenotype of the proband. All male patients presented with everted lipsat birth, especially at the lip corners (**b**), genu varum with unknown cause after starting to walk (**c**), and cubitus valgus (**d**). **e** Whole genome linkage analysis showing a 4 Mb critical region on chromosome X. **f** Schematic diagram showing RefSeq genes in the critical region. A red bar indicates the position of the Xq27.1 palindrome

A five-generation Chinese family was identified with a distinct compound phenotype involving genu varum, cubitus valgus, and everted lips (Fig. 1a–d). The family included ten affected individuals, all males, consistent with X-linked recessive inheritance. After obtaining written informed consent from participants and approval from the Peking Union Medical College institutional review board, peripheral blood samples were collected from 28 family members, including 5 patients and 23 phenotypically normal members. Genomic DNA was extracted using a QIAamp DNA blood mini kit (Qiagen, Valencia, CA, USA) by standard methods according to the manufacturer’s instructions. Systemic physical examinations and accessorylab examinations of the proband (V25) were provided by relevant physicians.

### Genotyping and linkage analysis

Linkage analysis was performed on selected individuals from the family. Five affected (IV3, IV5, IV11, IV23, V25) and three unaffected members (III9, IV25, IV30) were genotyped using the Affymetrix Genome-Wide Human SNP Array 6.0, containing over 906,600 SNPs and 946,000 copy-number probes, following the manufacturer’s protocol. In brief, genomic DNA was digested with restriction endonuclease, ligated to the relevant adaptor, then PCR amplified using unique single primers. After fragmentation of the product with DNase I and terminal biotin labelling, sample hybridization was performed with the array and initial scanning data was acquired. Genotype calling, genotyping quality control, and CNV identification were performed using Affymetrix Genotyping Console 3.0 software. Non-parametric linkage analyses of the X chromosome was performed using MERLIN 1.1.2 to identify the potential linkage locus with the compound phenotype. Genotyping and data analyses were accomplished at CapitalBio Co., LTD (Beijing, China).

### Next-generation sequencing

Whole exome sequencing was conducted in the proband. Exome capture and sequencing was done by the Beijing Genomics Institute (BGI) (Shenzhen, China) using the NimbleGenSeqCap EZ Exome Library v2.0 (RocheNimbleGen, Madison, WI, USA)and HiSeq™ 2000 sequencing platform (Illumina, San Diego, CA, USA). Results were filtered to reduce the list to those restricted to the linkage region and predicted as damaging or possibly damaging by SIFT, PolyPhen-2 and with allele frequencies less than 0.1% in the 1000 genomes project and Exome variant server data, then validated by Sanger Sequencing and cosegregated with the phenotype.

The proband and his phenotypic-normal parents were analyzed by WGS using a NEBNextUltra II DNA Library Prepkit for Illumina (New England Biolabs, Ipswich, MA, USA) and a HiSeq X Ten sequencer (Illumina, San Diego, CA, USA). Sequencing covered more than 99% of the genome with an average of30 reads or more. > 86% of sequenced bases achieved quality scores higher than Q30. Reads were aligned to the GRCh37/hg19 human reference sequence using the Burrows–Wheeler Aligner (BWA, v.0.7.8-r455)and variant calling was performed with SAMtools (v.1.0) and annotated using ANNOVAR (v.2015Dec14). Picard (v.1.111) was used to merge BAM files of the same sample and filter out duplicate reads marked. SNP/Indel, CNV, and SV variants were called and classified by SAMtools (v.1.0), Control-FREEC (v.V7.0), and CREST (v.V0.0.1), respectively. Candidate variants were scanned among the refined linkage region in chromosome X and further confirmed by Sanger Sequencing and cosegregation analysis in the pedigree.

### Gap-PCR and genome walking

To confirm the rearrangement in chromosome X, gap-PCR and genome walking assays were designed. A 573 bp PCR product was amplified by gap-PCR according to the implicated breakpoints in WGS, and distal breakpoint junctions were validated by Sanger Sequencing. Chromosomal walking from the known sequence distal to the palindrome was performed with a Genome Walking Kit (Takara Bio., Dalian, China) following the recommended protocol using common partially degenerated primers and a set of nested locus-specific primers to define the origin of the insertion fragment in successive rounds of thermal asymmetric interlaced PCR (TAIL-PCR). The proximal breakpoint junction was amplified and detected by gap-PCR using specific primer pairs. Primer sequences are listed in Additional file 1: Table S1.

### qPCR assays

Real-time quantitative PCR (qPCR) assays were designed to detect copy number changes in the candidate region suggested by the Affymetrix Genome-Wide Human SNP Array 6.0. Three pairs of primers were designed within the duplicated region and one pair was designed spanning the breakpoint chrX: 733,365 implicated in WGS. Primer sequences are listed in Additional file 1: Table S1. qPCR assays were run on a Rotor-gene Q/RG-6000system (Qiagen, Hilden, Germany). Relative copy number (RCN) was determined with the comparative ΔΔCTmethod, and a ~ 1.5-fold RCN was used for duplication. Four family members with sufficient DNA samples were analyzed: III9 (female carrier), IV5 (affected male), V25 (affected male), and IV25 (unaffected male). One unrelated female and one unrelated male were analyzed as controls.

### RT-PCR assays

Conventional RT-PCR was used to detect the expression of *FGF13*, *SOX3*, *FGF13*-*AS1*, and the housekeeping gene *ACTB*in the peripheral blood of the proband and unrelated male and female controls using primers shown in Additional file 1: Table S1.

## Results

### Phenotype description

All patients in the family presented a characteristic lip shape at birth, with fish mouth-like, everted, thick lips, notably at the lip corners (Fig. 1b). No obvious abnormality of the arms, legs, or knee or elbow joints was observed at birth or before 1 year of age. After starting to walk, genu varum presented and became more serious with age, resulting in limited daily activities. The proband (V25) was the first child of his non-consanguineous parents, full-term normal delivery, birth weight 3.5 kg, length 50 cm. Everted lips were observed at birth. Developmental milestones were normal reached. Genu varum appeared when he started to walk at 1-year-old and became worse as height and weight increase. He received a high tibial osteotomy at age ten but the genu varum was not corrected. The space between the proband’s knees was 15 cm while standing naturally (Fig. 1c). This reduced significantly when standing forcibly by himself. Cubitus valgus was also noticed (Fig. 1d). He was 19 years old when he first came to our hospital, with height 165 cm and weight 52 kg. No dysmorphic feature was observed in his head, chest or spine. Psychomotor and intellectual development is normal. Physical examination showed muscle strength of V grade and normal muscle tension. No abnormality of bone metabolism was found by laboratory examination, with serum calcium 2.44 mmol/L, serum phosphate 1.4 mmol/L, serum alkaline phosphatase 46 U/L, ionized calcium 1.12 mmol/L, PTH 25.9 pg/mL, 24-h urine calcium 3.88 mmol, 24-h urine phosphate 4.86 mmol, 1,25(OH)_2_D_3_ 51.17 pg/mL, and β-CTX 0.2 ng/mL. X-ray examination showed wide interspacing of the knee joint and patellar dislocation, and no obvious changes were observed in sclerotin, bone cortex, or bone trabecula of the bones composing the hip and knee joints (Additional file 2: Figure S1).

### Disease locus mapping and coding region mutation screening in chromosome X

To determine the causative locus, linkage analysis and fine mapping was performed in the family. A candidate interval of 4 Mb (chrX: 136,218,962–140,388,078, hg19) containing 20 RefSeq genes, with LOD > 2, was identified (Fig. 1e, f). Whole exome sequencing was then performed in the proband to detect mutations in the coding region, especially in the candidate 4 Mb interval. Detected coding SNPs and small indels were sequenced in all available family members, yet no coding region mutations fully co-segregated with the phenotype in the family.

### Structure variation and noncoding region mutational detection

To determine whether the rare X-linked compound syndrome was caused by an unknown microdeletion or microduplication, we performed a genome-wide high-resolution CNV scan. We did not detect any potential pathogenic CNVs which are absent in the DGV database in the critical region. A trio in the family (V25, IV29, and IV30) underwent WGS to detect complicated structural variation and SNPs or indels in the noncoding region. An average of 99.97 Gb of sequence was generated with a mean coverage depth of 34.54× across the whole genome for each individual. WGS of the trio implicated an intra-chromosomal translocation with one breakpoint embedded in the critical region. Two breakpoints implicated in WGS are at chrX: 733,365 and chrX: 139,502,956. The chrX: 139,502,956 breakpoint within the critical region was supported by four split reads with coverage of five in the male patient, two split reads with coverage of 12 in the obligate carrier mother, and no split reads with coverage of 13 in the unaffected father. The other breakpoint chrX: 733,365 outside the critical region, was supported by five split reads with coverage of 52 in the male patient, ten split reads with coverage of 44 in the obligate carrier mother, and no split reads supported in the unaffected father.

### Identification of an insertion into the Xq27.1 palindrome from PAR1

**Fig. 2:**
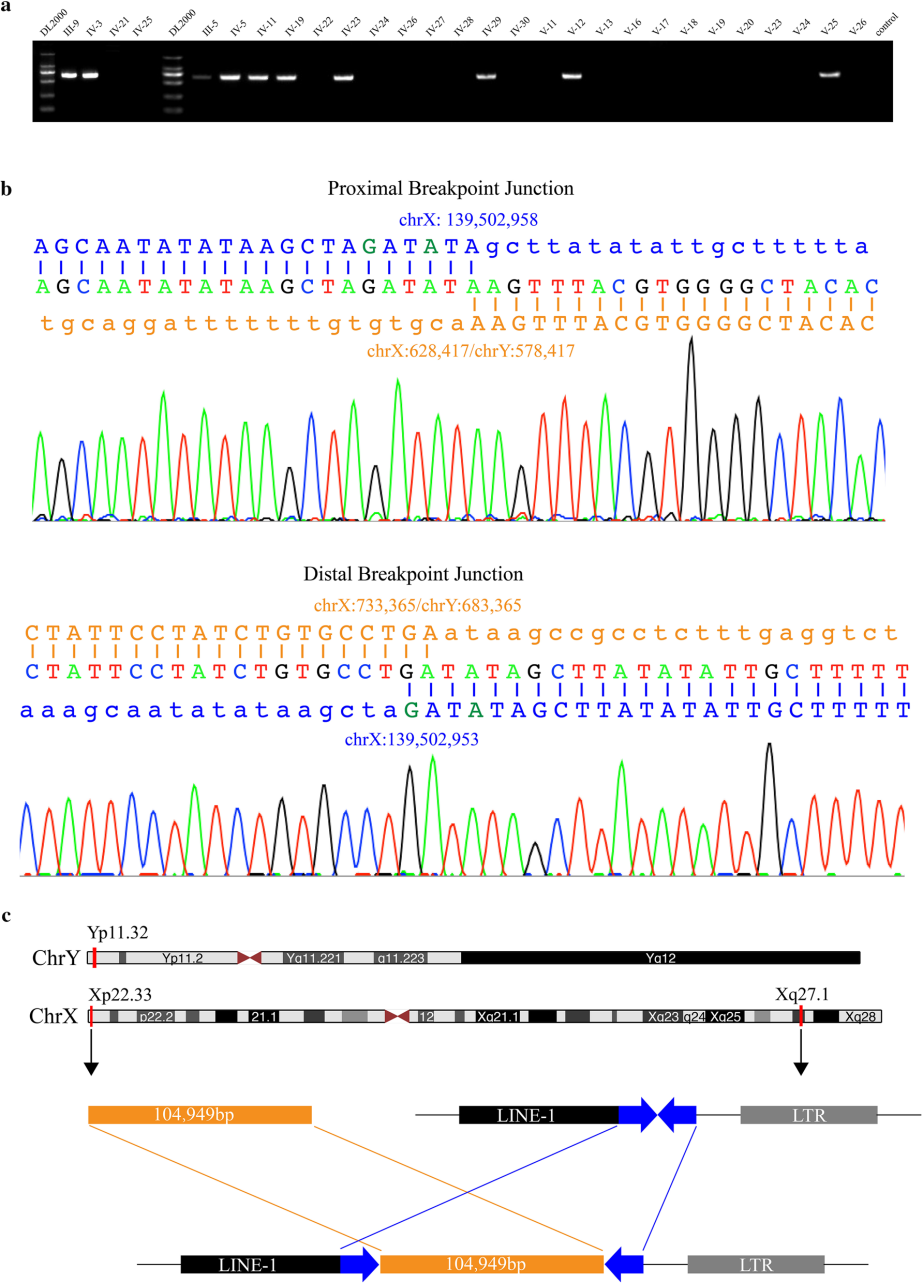
Identification of an inherited interstitial insertion at Xq27.1 in the Chinese family with a rare X-linked recessive compound phenotype. **a** A 573 bp gap-PCR product of the distal breakpoint junction showing segregation with the phenotype in the family. **b** Chromatogram of the proximal (upper) and distal (lower) breakpoint junctions. Reference sequences on Xq27.1 and pseudo-autosomal region 1 (PAR1) are indicated in blue and orange, respectively. Minimal sequence homology of “A” and “GA” are observed at the breakpoint junctions. **c** Schematic diagram of the identified interstitial insertion in the Xq27.1 palindrome. Orange solid bar represents the 105 kb inserted fragments from the pseudo-autosomal region Xp22.33/Yp11.32. Blue head-to-head arrows represent the 180 bp human specific palindrome at Xq27.1 flanked by long interspersed elements-1 (LINE1) and long terminal repeat (LTR) sequences

**Fig. 3:**
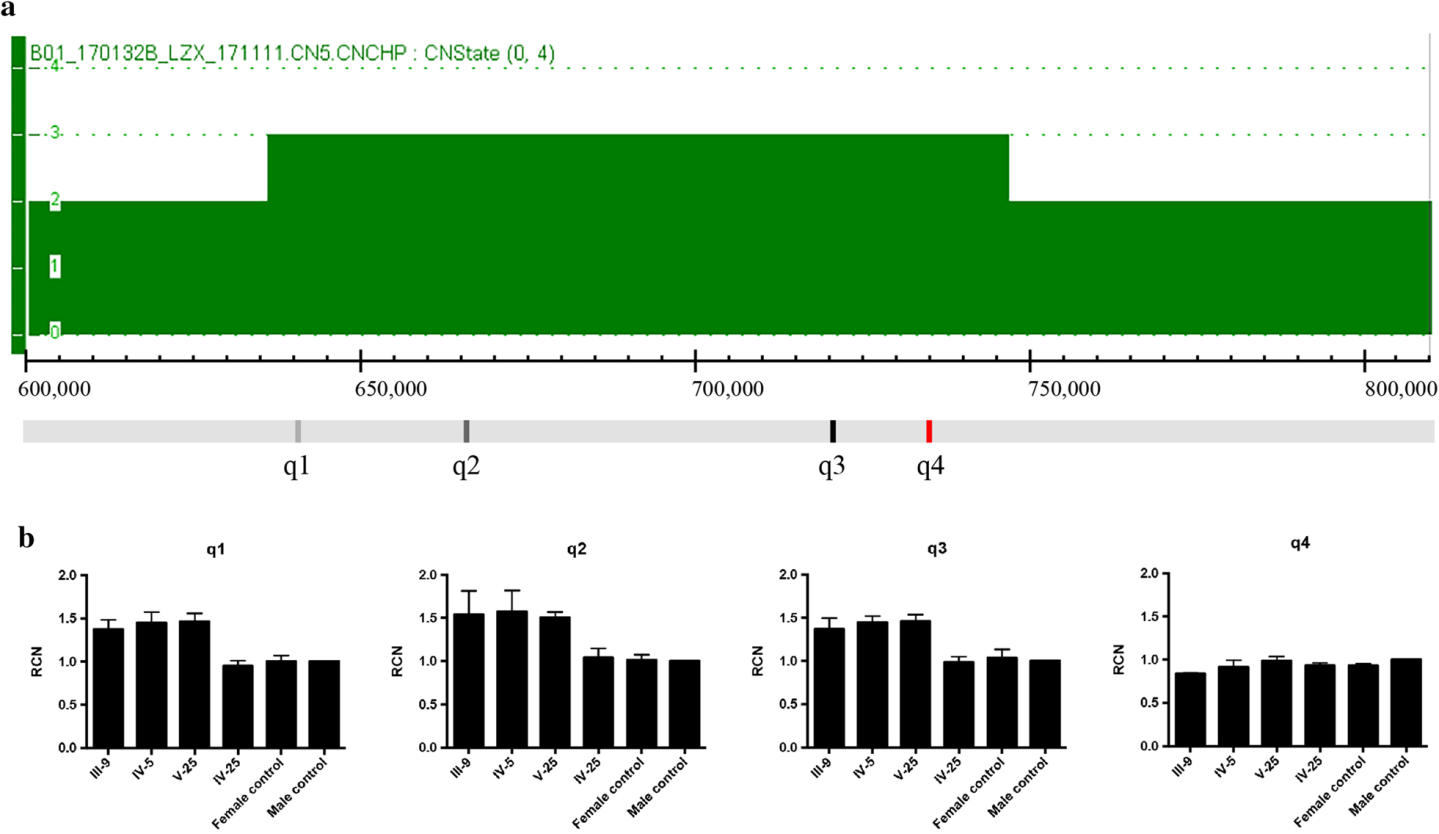
Identification of a 105 kb duplication of pseudo-autosomal region (PAR) of sex chromosomes. **a** SNP array analysis showing a copy number gain at the Xp22.3, homologous with Yp11.32, in male patients. **b** Confirmation of the duplication by qPCR assays. Positions of four designed qPCR assays are indicated at the bottom of **a** (black bars). Three qPCR assays within the indicated duplicated region (q1, q2 and q3) confirmed one copy number gain in male patients and female carriers in family, while qPCR with primers across the breakpoint (q4) showed no copy number changes in the family

To validate the identified structural variation, gap-PCR was performed spanning the two breakpoints. A 573 bp gap-PCR product was detected in all male patients and obligate female carriers in the family (Fig. 2a). Sanger Sequencing of the generated PCR products confirmed the junction of two breakpoints, considered to be the distal breakpoint junction (Fig. 2b). Genome walking from the locus-specific sequence distal to the breakpoint chrX: 139,502,956 also revealed the junction of two breakpoints. We noticed the chrX: 139,502,956 breakpoint was near the center of the palindromic sequence at Xq27.1, while the chrX: 733,365 breakpoint was located at Xp22.33, homologous with Yp11.32, in the pseudoautosomal region 1 (PAR1) of the sex chromosomes. CNVs in PAR were easily missed due to homology between the X and Y chromosomes, so we re-evaluated the SNP array data and found a > 100 kb copy number gain involving the identified chrX: 733,365 breakpoint in PAR1 in the proband (Fig. 3a). Copy number gains in the same region were also observed in four affected males (IV3, IV5, IV11 and IV23) and one obligate female carrier (III9), but not in unaffected familial males (IV25 and IV30) or 42 unrelated controls. To confirm this, we designed four qPCR assays to detect CNVs in four informative family members and two unrelated controls. Three qPCR assays within the implicated duplication region confirmed a copy number gain in the male patients and female carriers in the family, while one qPCR assay with primers spanning the breakpoints showed no CNV changes in the family (Fig. 3b). Combining qPCR and SNP array data, a ~ 105 kb duplication in PAR1 was confirmed with full co-segregation with the disease phenotype in the family. Considering the breakpoint junction identified by WGS, we speculated that the duplicated PAR1 fragment could insert in the palindrome at Xq27.1 (Fig. 2c). We further amplified the breakpoint junctions with primers from the sequences flanking the palindrome at Xq27.1 and the boundary of the Xp22.33 duplication. Sequence analysis of the resultant amplicons by Sanger sequencing verified the distal junction pinpointed in WGS (chrX: 733,365/chrY: 683,365 and chrX: 139,502,956) and showed the proximal junction between the lower boundary of the Xp22.33 duplication and the Xq27.1 palindrome (chrX: 628,417/chrY: 578,417 and chrX: 139,502,956; Fig. 2b). The junction sequencing results also indicated the duplicated fragment was inserted into Xq27.1 palindrome indirect orientation.

### Expression detection of nearby genes

Since the 105 kb insertion contained no coding genes, we presumed the insertion could impact genes near the Xq27.1 palindrome via positional effects. We tested expression levels of three candidate genes within a topological associated domain on Xq27.1. As a result, we detected no expression of *FGF13*, *SOX3*, or *FGF13*-*AS1* in peripheral blood of the proband or in unrelated controls.

## Discussion

An rare X-linked recessive inherited compound phenotype is reported in the present study. It is characterized by genu varum, cubitus valgus, and a carp-shaped mouth, and is transmitted in an X-linked recessive manner in a five-generation Chinese family. All male patients in the family presented with a thick, carp-shaped mouth at birth, such that family members could infer whether the newborn boy would develop genu varum. As patients grew and developed, joint dysfunction gradually appeared, manifested by limited elbow rotation and excessive relaxation of the knee joint leading to genu varum. We reviewed all 341 phenotypic entries on chromosome X in the OMIM database and found none matching the identified phenotype. Among all the presenting phenotypes, the knee joint malformation leading to limited activities most seriously affected the patients’ normal life. Genu varum is seen in various metabolic and developmental bone diseases such as hypophosphatemic rickets, metaphyseal chondrodysplasia, and Blount disease. Clinically, a blood biochemical examination and typical radiographic features can be used for differential diagnosis of rickets and metaphyseal chondrodysplasia [15]. Patients in the family showed no abnormality of bone mineralization via blood biochemical examination and no obvious changes were observed in sclerotin, bone cortex, or bone trabecula of bones composing the hip and knee joints, ruling out rickets and metaphyseal chondrodysplasia. The proband was initially diagnosed with Blount disease in another hospital and received surgical intervention at the age of ten, though the symptoms were not relieved. Blount disease is a developmental condition characterized by disordered endochondral ossification of the medial part of the proximal tibial physis, resulting in multiplanar deformities of the lower limb causing patients to become bowlegged. The cause is unknown, but it tends to run in families. Review of the patient’s X-ray results showed no typical radiographic features of Blount disease, with the tibial shaft in the varus position, a wedge-shaped epiphysis, a depressed adjacent metaphysis, a beak-like protuberance of rarified bone, and increased metaphyseal-diaphyseal angle. Thus, the phenotype is not consisted with Blount disease. The physiological structure and stability of knee joints are maintained by osseous structure and surrounding soft tissue including synovium, the joint capsule, and ligaments [16, 17]. Since we saw no evidence of osseous structure abnormality in the patients, we suspect that there might be a problem with their soft tissue. Further evidence should be obtained from pathological examination of the surrounding soft tissue such as skeletal muscle and ligament.

Although the pathological changes have not been elucidated, the underlying genetic alteration has been detected. After a full set of genome-scale mutational screening including whole genome linkage analysis, WES, WGS, and whole genome CNV scan, we identified a 105 kb interstitial insertion into the human-specific Xq27.1 palindromic sequence with full cosegregation with the rare phenotype. The human-specific Xq27.1 palindrome isprone to breakage and appears to be a hotspot for genomic rearrangement. Several human diseases/phenotypes associated with the Xq27.1 palindromic insertion have been reported, including hypertrichosis, hypoparathyroidism, ptosis, sex reversal, and Charcot–Marie–Tooth disease (Table 1). These reported cases are clinically distinct, even though they have nearly identical X chromosome breakpoints within the 180 bp palindromic sequence. Therefore, the different phenotypes are associated with the inserted genomic fragments, not with the insertion position itself. There are several possible hypotheses of the underlying mechanism, the first being overexpression of genes within the insertion fragments due to the trisomy of complete or partial gene transcripts. The previously reported inserted fragments are all from autosomal chromosomes and all contain complete or partial coding gene/s. However, none of the genes in the reported cases have been implicated in the corresponding phenotypes. In the present study, the 105 kb duplication is from the pseudoautosomal region of the sex chromosomes. No coding genes were in the duplicated genomic fragment. This provides direct evidence that the Xq27.1 associated phenotypes are not due to trisomy of coding genes in the insertion fragments. The second hypothesis is that large insertions in the Xq27.1 palindrome may have positional effects on the expression of nearby genes. DeStefano et al. suggested a positional effect may lead to altered *FGF13* expression in affected hair follicles and the terminal hair overgrowth phenotype of X-linked hypertrichosis [7]. However, this is unlikely to be a common mechanism underlying all Xq27.1-associated phenotypes, since not all Xq27.1 insertion carriers have hypertrichosis, especially those with insertions larger than 386 kb. The third hypothesis is that the fragments may contain regulatory elements, such as promoters, enhancers, and silencers, leading to transcriptional dysregulation of one or more genes nearby the Xq27.1 palindrome. We are inclined to agree with this hypothesis because three previous studies showed direct evidence of dysregulation of nearby *SOX3* or *FGF13*, both of which are important developmental regulators, in Xq27.1 palindromic insertion carriers [6–8]. There are also evolutionally conserved regions and predicted regulatory elements from ENCODE (encyclopedia of DNA elements) data in the identified 105 kb duplicated region (Additional file 3: Figure S2). We tested the expression of *SOX3*, *FGF13*, and *FGF13*-*AS1* by qPCR in peripheral blood from the proband. No expression of these genes was detected in the proband or unrelated controls. We postulate that the regulatory elements introduced by the insertions are spatiotemporally specific or that other genes in the same topological associated domain are ectopically expressed.

**Table 1:** Human diseases/phenotypes associated with Xq27.1 palindromic insertions

Diseases/phenotypes	Insertion origin	Insertion size	Genes within insertion	Insertion direction	Abnormal expressed genes	References
Hypoparathyroidism	2p25.3	305–340 kb	*SNTG2*	Direct	ND	Bowl et al. [5]
Congenital generalized hypertrichosis	5q35.3	126 kb	*COL23A*	Direct	ND	Zhu et al. [4]
Congenital generalized hypertrichosis	4q31.2	300 kb	*PRMT10*, *TMEM184C*; *ARHGAP10*, *EDNRA*	Inverted	ND	Zhu et al. [4]
Congenital generalized hypertrichosis	6p21.2 and 3q21.1	386 kb and 56 bp	*DAAM*, *KIF6* and *FAM162A*	Inverted	Decreased expression of *FGF13* in skin	DeStefano et al. [7]
Congenital bilateral isolated ptosis	1p21.3	120 kb	*DPYD*	Direct	ND	Bunyan et al. [9]
SRY-negative XX male sex reversal	1q25.2–25.3	774 kb	*ACBD6*, *XPR1*, *KIAA1614*, *STX6*, *OVAAL*, *MIR3121*, *LHX4* and *MR1*	Direct	Increased expression of *SOX3*	Haines et al. [8]
Charcot–Marie–Tooth neuropathy CMTX3	8q24.3	78 kb	*ARHGAP39*	Direct	Increased expression of *FGF13*	Brewer et al. [6]
X-linked recessive genu varum, cubitus valgus and characterized lip shape	Xp22.3/Yp11.32	105 kb	None	Direct	ND	Present study

*ND* not detected

The 180 bp palindromic sequence on Xq27.1 is evolutionarily young. Only humans have the entire palindromic sequence with both halves replicated exactly in the opposite direction. Chimps also have the two halves of the palindrome, but in the same direction, and other nonhuman primates have only one half of the palindrome [4]. The human-specific palindromic sequence has potential to form hairpin loops, which are susceptible to double-stranded DNA breaks [18]. The palindrome is also flanked by a LINE-1 repeat and an LTR sequence [19], which are both mediators of translocations. These sequence features suggest that the genomic region is highly unstable and might induce genomic rearrangement. This is also supported by the observation that all reported Xq27.1 palindrome-mediated insertions have at least one breakpoint near the center of the palindromic sequence where the hairpins form. The Xq27.1-mediated insertion is extremely rare, requiring not only breakage of the Xq27.1 palindrome but also incorrect repair from the duplicated genomic region. Brewer et al. proposed that this recurrent condition was initiated by the hairpin formation of the palindrome sequence and endonuclease activity, followed by microhomology-mediated break-induced replication (MMBIR) from nearby single-stranded DNA [6]. MMBIR is a replication-based mechanism of recombination between sequences with very little base identity [20]. Consistent with the MMBIR mechanism, we observed minimal sequence homology of 1 bp and 2 bp at the proximal and distal breakpoint junctions, respectively. MMBIR often couples with fork stalling and template switching (FoSTeS), a replicative mechanism for changing chromosome structure [21, 22]. All reported insertions were from different autosomal chromosomes, while our case was from the same chromosome but pericentric (on the other arm) to Xq27.1. These insertions were apparently ‘templated’ from nearby genomic intervals and were consistent with such rearrangements being generated by a DNA replication mechanism.

Trio sequencing for idiopathic phenotypes is highly practical and becoming more routine in clinical practice. In the case of this study, although one breakpoint junction was implicated in the trio sequencing results, the interstitial insertion could have been missed lacking the linkage information. Because the distinctive palindromic feature of sequence around the breakpoint would cause problems in alignment and result in low detection rate of WGS. Thus, for those “next generation sequencing-negative” families, the linkage data from a multi-generational family is very important, and sequence feature around the breakpoints should also be notice.

## Conclusions

We report an rare pedigree with a novel X-linked recessive compound phenotype caused by a 105 kb interstitial insertion into the Xq27.1 palindrome from PAR1. The present study provides an etiological diagnosis for the family and expands the spectrum of known human-specific Xq27.1 palindrome insertion events and associated phenotypes.

## Additional files

**Additional file 1: Table S1.** Primers used for gap-PCR, genome walking, qPCR and RT-PCR.

**Additional file 2: Figure S1.** X-ray photos of hip and knee joints. Photos of left knee joint (A, B), hip joint (C), and right knee joint (D). X-ray examination showed wider interspacing of the knee joint and patellar dislocation. No obvious changes were observed in sclerotin, bone cortex, or bone trabecula of bones composing the hip or knee joints.

**Additional file 3: Figure S2.** Characterization of the 105 kb duplicated region of PAR1. Evolutionarily conserved regions and predicted regulatory elements were observed within the duplicated region. Notice the track “100 vertebrates Basewise Conservation by Phylop”, “Transcription Factor ChIP-seq (161 factors from ENCODE with Factorbook Motifs)”, and “Chromatin State Segmentation by HMM from ENCODE/Broad” of nine types of cell.

## Abbreviations

PARs: pseudoautosomal regions
kb: kilobases
Mb: megabases
LCRs: low copy repeats
LINE1: long interspersed elements-1
LTR: long terminal repeat
CNVs: copy number variations
SVs: structure variation
qPCR: quantitative PCR
WES: whole exome sequencing
WGS: whole genome sequencing
ENCODE: encyclopedia of DNA elements
MMBIR: microhomology-mediated break-induced replication
FoSTeS: fork stalling and template switching
