# Supplementary material for: A 105 kb interstitial insertion in the Xq27.1 palindrome from pseudoautosomal region PAR1 causes a novel X-linked recessive compound phenotype
Source: J Transl Med. 2019 Apr 29;17:138. doi: 10.1186/s12967-019-1887-2 (PMC6489244; doi:10.1186/s12967-019-1887-2)
Supplement: Supplementary file 1 — Additional file 1: Table S1. Primers used for gap-PCR, genome walking, qPCR and RT-PCR. [file 12967_2019_1887_MOESM1_ESM.docx]

Additional file 1: Table S1. Primers used for gap-PCR, genome walking, qPCR and RT-PCR

| Primers | Sequences (5’ to 3’) | Application |
| --- | --- | --- |
| GP-D-F | TTCCATTGCAGGAGTGATGA | Gap-PCR for Distal breakpoint junction |
| GP-D-R | GCTTCCCTCTAAGCCCCTAA |  |
| GP-P-F | CTGCCATTGAAA-TCAACCAC | Gap-PCR for Proximal breakpoint junction |
| GP-P-R | AATAACAGGTTGCACCGAGGTT |  |
| GW-SP1 | CAATGTGGCCCATGAACTTCTTC | First-round Genome walking |
| GW-SP2 | TGGATTAGTGTTCTCCCACAGGA |  |
| GW-SP3 | GGATTTTAAAGGTGGCAGCATG |  |
| GW-SP4 | ATATAAGCTATATCAGGCACAGATAGG | Second-round Genome walking |
| GW-SP5 | CATCTGAACATTGCCCTGAAACAG |  |
| GW-SP6 | TCACTCCTGCAATGGAAACTTCAC |  |
| Q1-F | TGTCTGCACCAGAAAGATGG | qPCR within duplicated region |
| Q1-R | ACCTTCCACGTTGAAACCAG |  |
| Q2-F | CTTTGCTTGCAACAGCTCAG | qPCR within duplicated region |
| Q2-R | CAGGGGAACACCTCACTTGT |  |
| Q3-F | TGGAGTTTCTGCTGCTCTGA | qPCR within duplicated region |
| Q3-R | AGGTCTGTGGGAACTGCATC |  |
| Q4-F | TGGCTTTGCTATTCCTATCTGTG | qPCR spanning the breakpoint |
| Q4-R | GGATTGTTGAAAGAATGGTTGACAC |  |
| FGF13-F | AACCTCATCCCTGTGGGTCT | RT-PCR for *FGF13* expression |
| FGF13-R | GGTGTGAAAAGTTCCGAGGT |  |
| SOX3-F | AGACCAGGACCGTGTGAAAC | RT-PCR for *SOX3* expression |
| SOX3-R | AATTGTGCATCTTGGGGTTC |  |
| FGF13-AS1-F | CGCGCAGAGGTGCTTAAAAT | RT-PCR for *FGF13-AS1* expression |
| FGF13-AS1-R | AGCTCCTCTGAGGCCTTCTC |  |
| ACTB-F | TGGCACCCAGCACAATGAA | RT-PCR for *ACTB* expression |
| ACTB-R | CTAAGTCATAGTCCGCCTAGAAGC |  |
