# Supplementary figures and images for: A 105 kb interstitial insertion in the Xq27.1 palindrome from pseudoautosomal region PAR1 causes a novel X-linked recessive compound phenotype
Source: J Transl Med. 2019 Apr 29;17:138. doi: 10.1186/s12967-019-1887-2 (PMC6489244; doi:10.1186/s12967-019-1887-2)

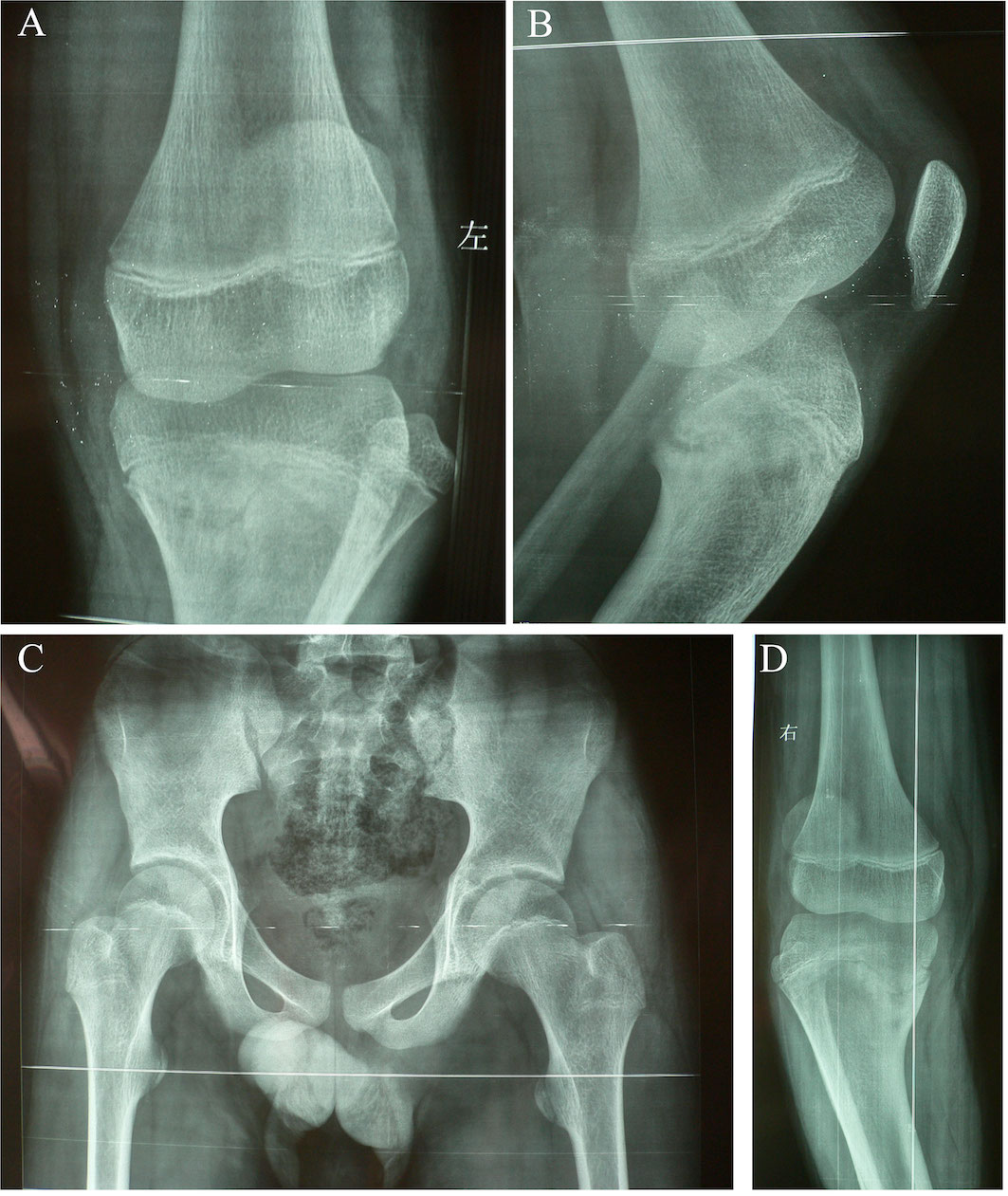

Supplement: Supplementary file 2 — Additional file 2: Figure S1. X-ray photos of hip and knee joints. Photos of left knee joint (A, B), hip joint (C), and right knee joint (D). X-ray examination showed wider interspacing of the knee joint and patellar dislocation. No obvious changes were observed in sclerotin, bone cortex, or bone trabecula of bones composing the hip or knee joints. [file 12967_2019_1887_MOESM2_ESM.jpg]

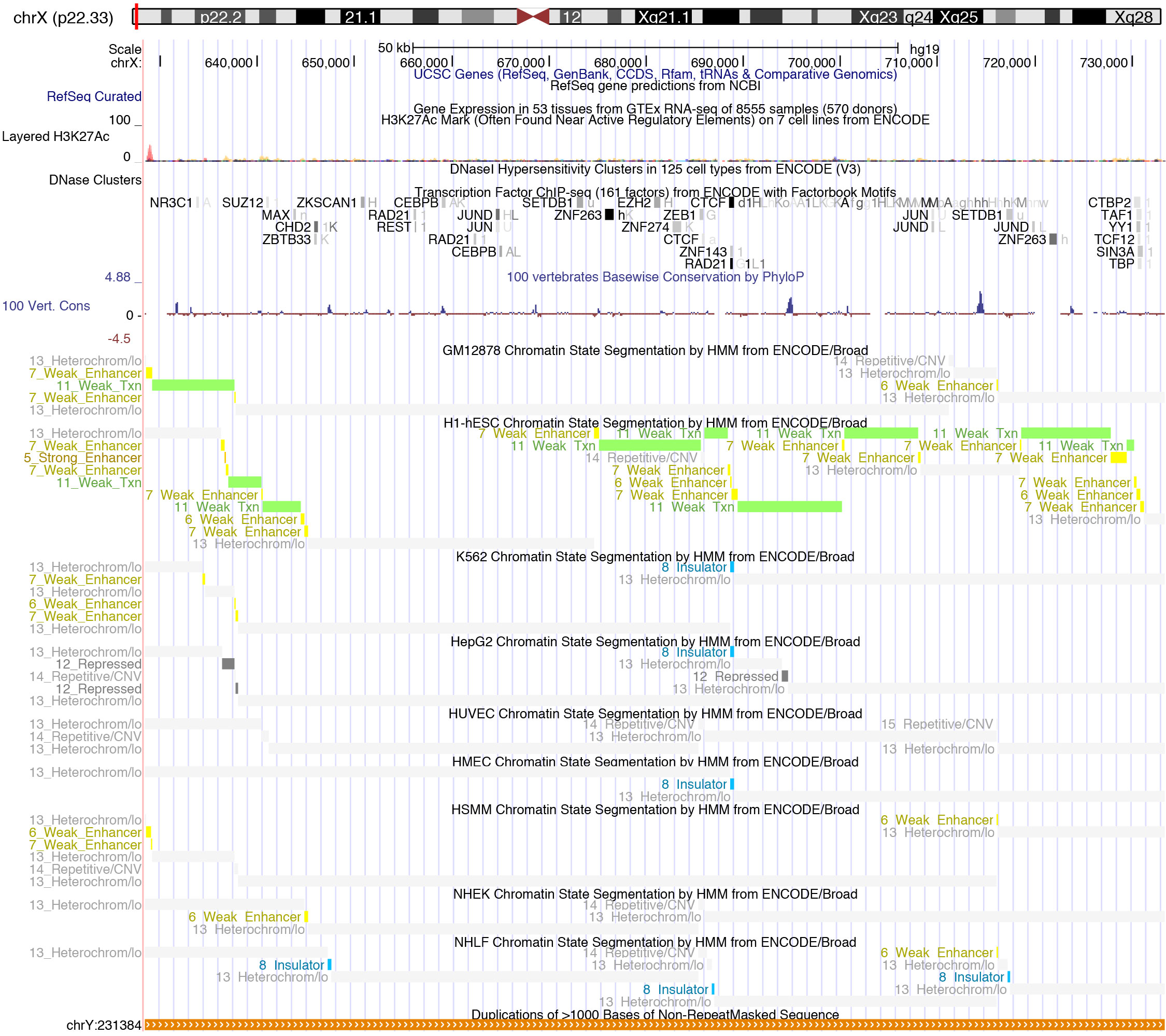

Supplement: Supplementary file 3 — Additional file 3: Figure S2. Characterization of the 105 kb duplicated region of PAR1. Evolutionarily conserved regions and predicted regulatory elements were observed within the duplicated region. Notice the track “100 vertebrates Basewise Conservation by Phylop”, “Transcription Factor ChIP-seq (161 factors from ENCODE with Factorbook Motifs)”, and “Chromatin State Segmentation by HMM from ENCODE/Broad” of nine types of cell. [file 12967_2019_1887_MOESM3_ESM.jpg]
